# Qi-Shen-Yi-Qi Dripping Pills Promote Angiogenesis of Ischemic Cardiac Microvascular Endothelial Cells by Regulating MicroRNA-223-3p Expression

**DOI:** 10.1155/2016/5057328

**Published:** 2016-02-15

**Authors:** Guo-Hua Dai, Ning Liu, Jing-Wei Zhu, Jing Yao, Chun Yang, Pei-Ze Ma, Xian-Bo Song

**Affiliations:** ^1^Department of Cardiology, Affiliated Hospital of Shandong University of Traditional Chinese Medicine, Jinan 250011, China; ^2^Department of Cardiology, Jining Hospital of Traditional Chinese Medicine, Jining 272000, China; ^3^Shandong University of Traditional Chinese Medicine, Jinan 250014, China; ^4^Department of Cardiology and Brain, Weihai Hospital of Traditional Chinese Medicine, Weihai 264200, China

## Abstract

Traditional Chinese medicine (TCM) research shows that Qi-Shen-Yi-Qi Dripping Pills (QSYQ) can promote ischemic cardiac angiogenesis. Studies have shown that microRNAs (miRNAs) are the key component of gene regulation networks, which play a vital role in angiogenesis and cardiovascular disease. Mechanisms involving miRNA by which TCM promotes ischemic cardiac angiogenesis have not been reported. We found that microRNA-223-3p (mir-223-3p) was the core miRNA of angiogenesis of rats ischemic cardiac microvascular endothelial cells (CMECs) and inhibited angiogenesis by affecting RPS6KB1/HIF-1*α* signal pathway in previous study. Based on the results, we observed biological characteristics and optimal dosage for QSYQ intervening in rats ischemic CMECs angiogenesis and concluded that QSYQ low-dose group had the strongest ability to promote angiogenesis of ischemic myocardium. Using miRNA chip and real-time PCR techniques in this study, we identified mir-223-3p as the pivotal miRNA in QSYQ that regulated angiogenesis of ischemic CMECs. From real-time PCR and western blot analysis, research showed that gene and protein expression of factors located RPS6KB1/HIF-1*α* signaling pathway, including HIF-1*α*, VEGF, MAPK, PI3K, and AKT, were significantly upregulated by QSYQ to regulate angiogenesis of ischemic CMECs. This study showed that QSYQ promote ischemic cardiac angiogenesis by downregulating mir-223-3p expression in rats ischemic CMECs.

## 1. Introduction

Ischemic heart disease is a common clinical condition with a high incidence rate. Currently, ischemic heart disease has been studied widely both in China and overseas in terms of means to promote ischemic cardiac angiogenesis. According to research in traditional Chinese medicine (TCM), the main causes for ischemic heart disease are a deficiency of Qi and clotting of blood vessels; therefore, TCM recommends Qi-replenishing and blood-activating therapy for treatment. “Qi-replenishing” is a method of nourishing vital essence, applying to shortness of breath, pale complexion, tired spirit, lassitude of limb, and other symptoms. Ancient Chinese literature has long recorded Qi-replenishing and blood-activating theories including “vasculogenesis” and “blood generating,” which are closely related to angiogenesis [1]. Qi-Shen-Yi-Qi Dripping Pills (QSYQ), comprising herbs such as* Astragalus*, red sage,* Panax notoginseng*, and* Dalbergia odorifera*, help replenish Qi, clear blood clots, activate blood flow, relieve pain, and are representative of Qi-replenishing and blood-activating TCM used to treat ischemic heart disease. MicroRNAs (miRNAs) are key regulators of gene expression, mainly via negative regulation, inhibiting target mRNA translation or inducing degradation of mRNA [2]. Thus, miRNAs play an important role in angiogenesis and cardiovascular disease. QSYQ can promote ischemic cardiac angiogenesis, but their mechanism of modulating ischemic cardiac angiogenesis by regulating miRNA expression has not been reported in detail.

In the previous study [3], we found that microRNA-223-3p (mir-223-3p) was the core miRNA of angiogenesis of rats ischemic cardiac microvascular endothelial cells (CMECs), which targeted* Rps6kb1* and inhibited angiogenesis of ischemic myocardium via regulating RPS6KB1/HIF-1*α* signal pathway. On the base, we focused on the biological characteristics and optimal dosage for QSYQ promoting rats ischemic CMECs angiogenesis. We utilized miRNA chips and real-time polymerase chain reaction (real-time PCR) techniques in this study, to analyze the mechanism through which QSYQ modulated angiogenesis by CMECs by regulating miRNA expression. It is concluded that QSYQ can downregulate mir-223-3p expression, activate RPS6KB1/HIF-1*α* signaling pathway, and promote ischemic cardiac angiogenesis.

## 2. Materials and Methods

### 2.1. Animal Gavage and Preparation of TCM-Containing Serum

This study was approved by the Committee of Ethics regarding Experimental Animals in Shandong Lukang Pharmaceutical Co., Ltd. A total of 20 male, Sprague Dawley rats (220 g to 280 g) were purchased from Animal Experiment Center of Shandong Lukang Pharmaceutical Co., Ltd., and randomly assigned to 4 groups: QSYQ high-dose group (Q-H), and 70 mg/(kg·d) was administered to it; QSYQ medium-dose group (Q-M), and 35 mg/(kg·d) was administered to it; QSYQ low-dose group (Q-L), and 17.5 mg/(kg·d) was administered to it; and blank serum group, and the same volume of distilled water was administered to it. Each group contained 5 rats and treatment was administered to each rat by oral gavage at 4 mL/treatment/day, for 5 days consecutively. An hour after the last gavage, serum was collected from the* aorta abdominalis *and prepared as per the method described below [4]. The rats were anesthetized via intraperitoneal injection of 10% chloral hydrate (0.3 mL/100 g), with limbs fixed and sterilized. Abdominal cavity was opened by a sterilized surgery scissor and the* aorta abdominalis* was isolated. A 0.2 mm gauge needle was inserted into the* aorta abdominalis* facing the direction of blood flow and blood was collected in a tube without anticoagulant. Rats were euthanized by overdose anesthesia with 10% chloral hydrate (0.6 mL/100 g) after harvesting 6–8 mL blood in the experiment. Blood was centrifuged for 15 min at 3000 rpm, serum was separated in a sterile environment, filtered through a 0.22 *μ*m microporous film, and aliquots were prepared, sealed, and stored at −20°C.

### 2.2. Rat Myocardial Infarction Model and Cell Culture

A model for myocardial infarction in rat was prepared as previously described [3], using the ligature method [5]. The ligature was considered successful when the postsurgery electrocardiogram showed Q wave and ST wave elevated as well as T wave elevated or upside down. Animals that survived for 24 h after surgery showed the success of the model. CMECs were cultured as previously described [3] using the explant culture method [6]. When a large number of cells surrounded the tissue, it was removed, medium was replenished every 2 days, and cells were cultured until there is almost fusion between the growing cells. Previous studies have shown that cells cultured in this way were CMECs [7].

### 2.3. Experimental Groups

Rat ischemic CMECs were randomly assigned into 4 groups: Q-H, Q-M, Q-L, and ischemia model (M). Normal rat-originated passage-zero CMECs (N) were used as control group. Thus, 5 groups were assigned in total. When cells have grown until near fusion, they were digested by 0.25% Trypsin-EDTA, suspended in cell culture medium with 10% TCM-containing serum (Q-H, Q-M, and Q-L groups) or blank serum (M and N groups).

### 2.4. Cell Proliferation

Trypan blue colorimetric assay was used to measure cell proliferation [8–11]. Cell suspension was seeded into a 96-well plate at 4 × 10^3^ cells/well, 6 wells/group, and 1 control well (with the same volume of cell-free medium added). Cells were cultured routinely for 7 days and 6 wells were used for colorimetry each day. For the colorimetry assay, 20 *μ*L of MTT solution (5 mg/mL) was added to each well and incubated at 37°C for 4 h. Culture supernatant was dispensed into fresh wells, 150 *μ*L of dimethyl sulfoxide was added, shaken well for 10 min, and absorption was measured at 490 nm using an enzyme-linked immunosorbent assay reader. Proliferation rate and proliferation window period were calculated. The growth curve was plotted for time on the horizontal axis and absorption value on the vertical axis.

### 2.5. Cell Migration

Cell migration was measured by wound-healing experiments [12]. Cell suspension solution was seeded in a 24-well plate at 5 × 10^4^ cells/well. After the cells are attached, a “wound” was created by marking a “+” sign in the center at the bottom of each well using a sterile 10 *μ*L tip and the wound-healing capacity of cells was monitored after 0, 6, 12, 24, 48, and 72 h using an inverted microscope (50x), images were captured, and cells that had migrated were counted. A total of 3 samples were taken from each group, in triplicate. Migration percentage and migration window period were calculated.

### 2.6. Tube Structure Formation

CMEC tube structure formation was studied as previously described [13, 14] and the number of tubes formed was counted. Cell suspension was seeded into 6-well plates at 5 × 10^4^ cells/well, 6 wells/group, and tube formation was monitored under an inverted microscope (100x) after 24, 48, 72, and 96 hours. Tube formation was considered positive if the endothelial cells interconnected, forming a C-shape. Within each well, 3 areas of intensive tube formation were selected, and tubes were counted. A total of 10 images were randomly collected from each group and used to calculate the tube formation percentage and tube forming window period.

### 2.7. miRNA Gene Chip Analysis

As per the window period characteristics of ischemic CMEC angiogenesis in each group, the optimal dosage and window period were selected as the intervention group. Total RNA was collected from the cells in the intervention, M, and N groups. The ratio of OD_260 nm_/OD_280 nm_ was measured by ultraviolet-visible spectroscopy and total RNA content was determined by formaldehyde-denatured agarose electrophoresis. miRNA was marked and the sample was concentrated with miRCURYTM Array Power Labeling kit and Rneasy mini kit assays. The miRNA chip was hybridized with miRCURYTM Array microarray kit and Hybridization Chamber II assays. Images were scanned using Genepix 4000B and data collected was analyzed using Genepix Pro 6.0 software. Based on gene expression content, differences at ≥ twice the gene expression were used as standard and N group was used as control, differentially expressed miRNAs from the intervention and M groups were selected, including miRNAs that were upregulated or downregulated ≥ 2 times, and miRNAs with significantly different expression were selected. These miRNAs were then compared with those with significantly different expression in the intervention and M groups and the core miRNA in QSYQ regulating ischemic CMECs was identified. Chip hybridization and data analysis were conducted at Shanghai Kangcheng Bioengineering Co., Ltd.

### 2.8. Confirming Core miRNA with Real-Time PCR

Real-time PCR was used to test the expression of core miRNA in different groups of cells. Total RNA was extracted from samples in each group and RNA concentration and purity were measured by ultraviolet-visible spectroscopy. RNA was reverse-transcribed to cDNA following reverse-transcription kit assay instructions. Real-time PCR with all cDNA samples was run as follows: 95°C for 5 min; 95°C for 10 s, 60°C for 10 s, and 72°C for 10 s, for 45 cycles; 95°C for 5 s, 60°C for 1 min, 97°C for 5 s, and 40°C for 30 s. Data was analyzed by using 2^−ΔΔCt^ method.

### 2.9. Analysis of mRNA Expression of Related Genes in the RPS6KB1/HIF-1*α* Signaling Pathway Using Real-Time PCR

Real-time PCR was used to test mRNA expression of* Rps6kb1*, HIF-1*α*, VEGF, MAPK, PI3K, and AKT in each group (intervention group, M group, and N group).

### 2.10. Western-Blot Analysis

Total protein was extracted from samples in each group using a total protein extraction reagent and protein concentration was estimated using BCA method. Sample solution and prestained protein markers were loaded and proteins were separated by electrophoresis. A stack of filter paper, gel, and cellulose was prepared as per Bio-Rad protein transfer unit instructions, and proteins were transferred to the cellulose membrane, blocked for 1 h in 5% fat-free milk at room temperature. Primary antibody was added to the blocked film, so that antigen and antibody combined and incubated overnight at 4°C and then secondary antibody with HRP marker was added. Meanwhile *β*-actin antibody with HRP marker was used to test *β*-actin content. The substrate was added, after which the film combined and reacted with the chemiluminescent substrate, and images were captured, scanned, and analyzed to calculate the sample density with densitometry. Density of target protein divided by density of inner control *β*-actin, to adjust for standard error, was calculated, and the relative content of target protein in the sample was obtained.

### 2.11. Statistical Analysis

The data was presented as mean ± standard deviation. Statistical comparisons were performed using one-way analysis of variance and independent sample *t*-test after one-way ANOVA. *P* < 0.05 was considered statistically significant.

## 3. Results

### 3.1. Rat Myocardial Infarction Model Building and Cell Culture

**Figure 1:**
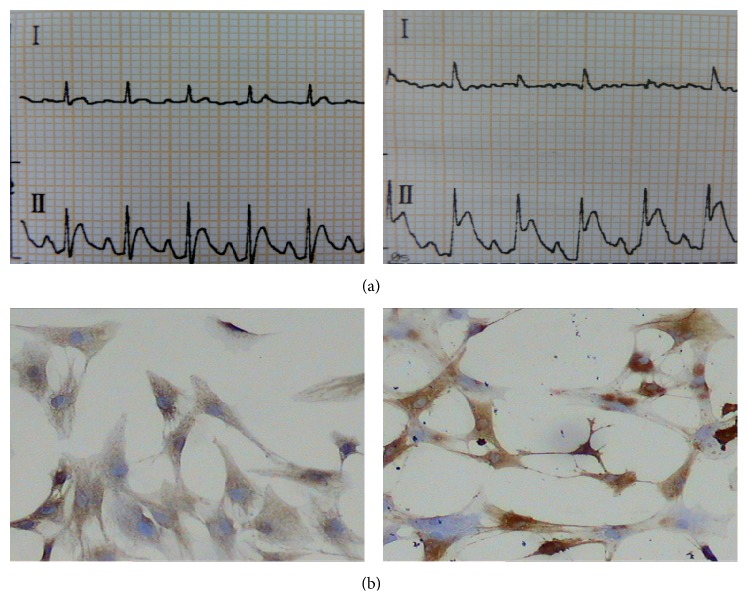
(a) left: a normal electrocardiogram of rat before the model building. (a) right: after the model building, electrocardiograms showed II indicated ST segment elevated significantly, which indicated ischemia myocardial and proved that the artery ligation was successful. (b) Immunocytochemical stain revealed that, after the staining of VIII factor (left), the cytoplasm was brown coloring and the coloring was the most significant in pericaryon and, after the staining of CD31 (right), their cell membrane showed yellowish brown particles, which proved that the cultured cells were CMECs.

This study had used 54 rats finally, and there were two rats that failed in respiration and circulation in 24 hours after mice model establishment. After the rat's left anterior descending coronary artery (LAD) was ligated, electrocardiograms indicated that ST segment elevated significantly (Figure 1(a)), which proved that the built model was successful. Immunocytochemical stain revealed that VIII factor and CD31 expressed positively in cells (Figure 1(b)), which proved that the cultured cells were CMECs.

### 3.2. Growth Curve and Proliferation Rate of Ischemic CMECs

**Figure 2:**
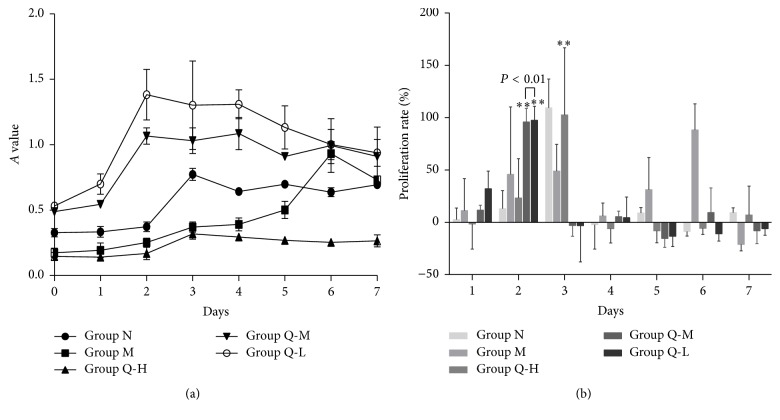
(a) The OD value of ischemic CMECs in Q-L group was significantly higher than that of other groups. Ischemic CMECs in Q-L group and Q-M group proliferated vigorously on the second day, while M group was on the sixth day. (b) A dynamic observation revealed that proliferation rate for all the three QSYQ-treated groups was higher than that of the M group (^*∗∗*^ *P* < 0.01). The proliferation window period of ischemic CMECs in Q-L group and Q-M group was four days ahead of the M group.

Latent periods during growth were observed for CMECs in each group. Rapid growth was noted on the 2nd day in Q-L and Q-M groups, compared to the 3rd day in the Q-H and N groups, after which a plateau was observed. M group showed rapid growth on the 6th day and did not show any obvious plateau (Figure 2(a)). Proliferation rate for the QSYQ-treated groups was higher than that for the M group, while proliferation rate of Q-L group was significantly higher than that of Q-M group (*P* < 0.01, Figure 2(b)). The window period of proliferation was found for cells within each group via active monitoring (Table 1).

### 3.3. Ischemic CMEC Migration and Tube Structure Formation

**Figure 3:**
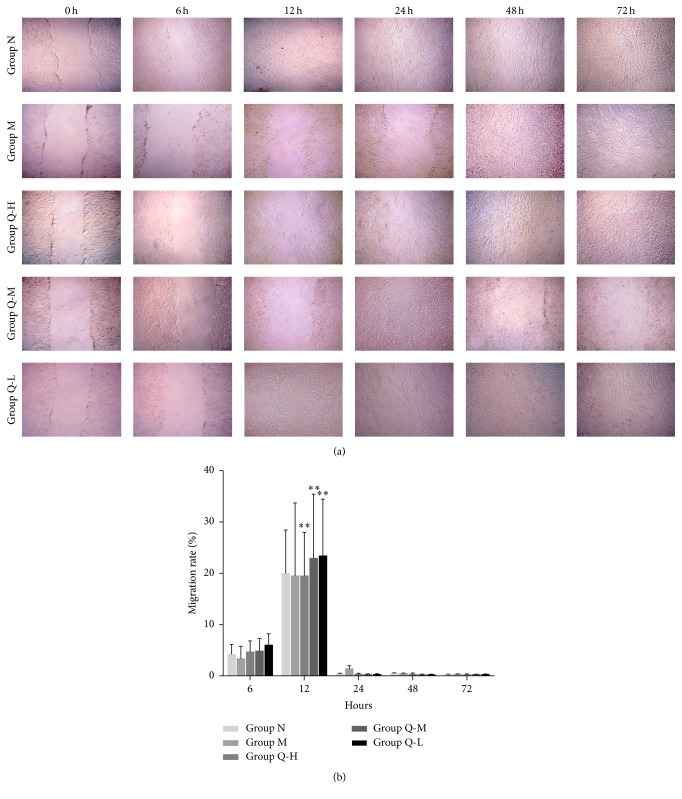
(a) For all groups, a clear scratching blank area could be spotted after scratching test zero hour under microscope (50x), while the blank area was covered extensively with the large amount of cells after twelve hours. The numbers of migrated cells in the QSYQ-treated groups were more than that in M group at the same timescale. (b) A dynamic observation revealed that migration rate of all the three QSYQ-treated groups increased significantly compared with that for the M group (^*∗∗*^ *P* < 0.01), especially Q-L group.

**Figure 4:**
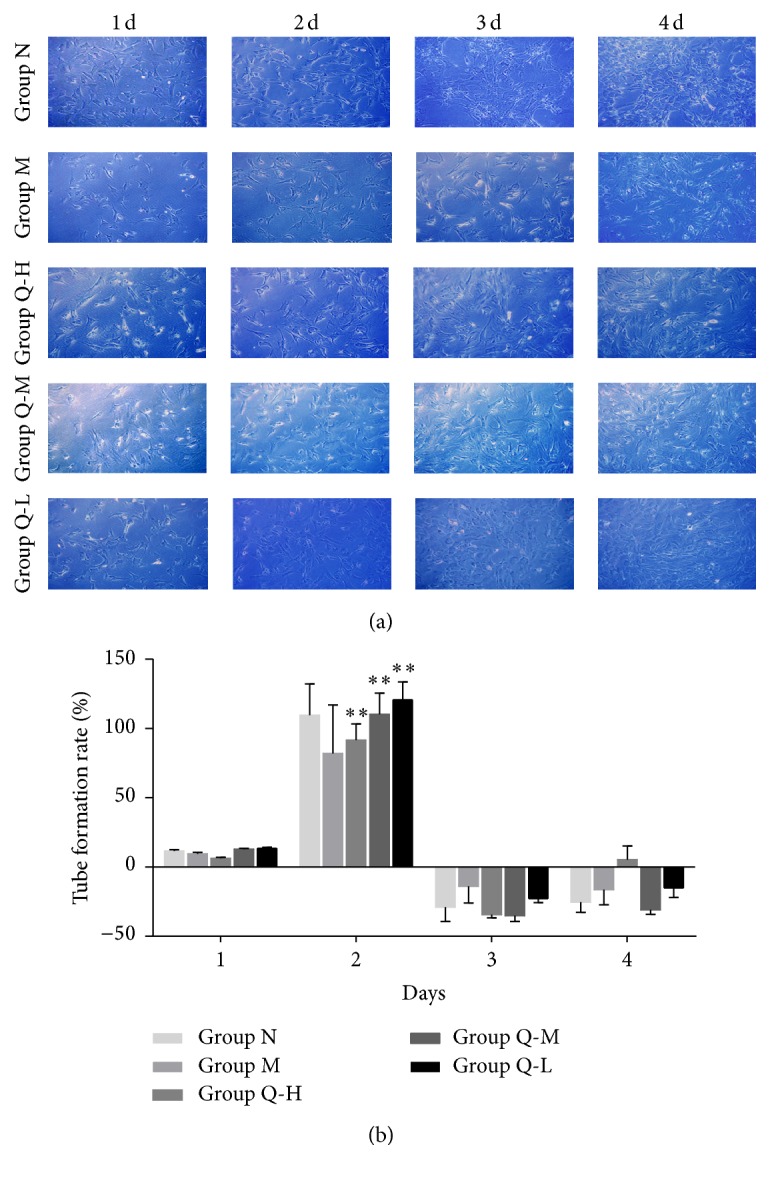
(a) For all groups, observation under inverted phase contrast microscope (100x) revealed that amounts of “C” shaped tube formation were formed significantly on the second day. The “C” shaped tube structure decreased gradually on the third day and the fourth day with increased cell numbers. The tube formation counts for the QSYQ-treated groups were more than that for the M group at the same timescale. (b) A dynamic observation revealed that tube formation rate of all the three QSYQ-treated groups increased significantly compared with that for the M group (^*∗∗*^ *P* < 0.01), especially Q-L group.

Compared with M group, significantly more cells migrated in the QSYQ-treated group (Figure 3(a)); the migration rate of Q-L group at 12 hours was significantly higher than that of other groups (*P* < 0.01, Figure 3(b)); the migration window period in each group was found via active monitoring (Table 1). Compared with M group, the tube formation numbers in QSYQ-treated groups were significantly higher (Figure 4(a)); tube formation rate of Q-L group on the 2nd day was significantly higher than that of other groups (*P* < 0.01, Figure 4(b)); the cell tube formation window period of each group was found via active monitoring (Table 1).

### 3.4. QSYQ Regulate miRNAs Expression during Ischemic CMECs Angiogenesis

**Figure 5:**
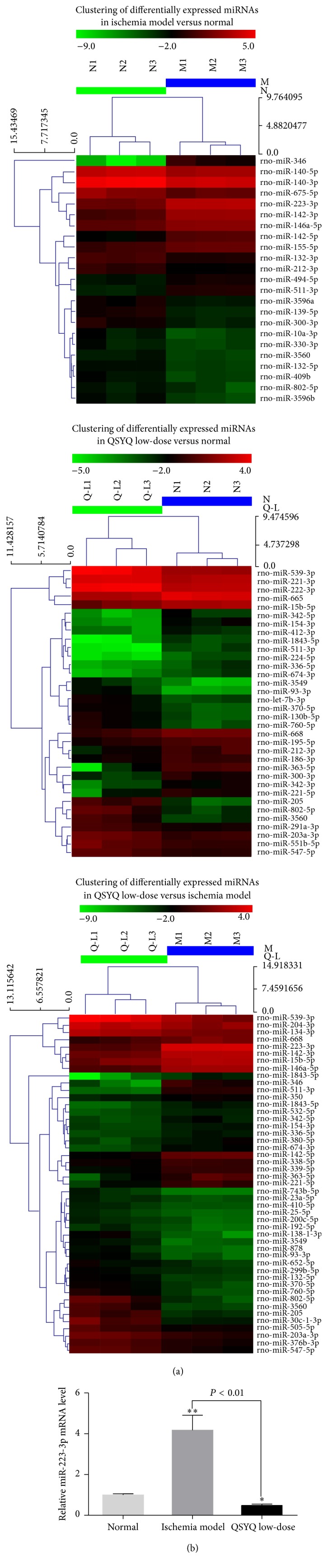
(a) The heat map diagram displayed the results of the two-way hierarchical clustering of miRNAs and samples. Each row represented a miRNA and each column stood for a sample. The miRNA clustering tree was displayed on the left, and the sample clustering tree appeared at the top. Cluster analysis arranged samples and miRNAs into groups based on their expression levels. Red indicated highly relative expression, and green indicated low relative expression. (b) The results of microarray assay of mir-223-3p were confirmed by real-time PCR in the same set of samples. ΔCt values were normalized to U6 levels. The results were expressed as 2^−ΔΔCt^. ^*∗*^ *P* < 0.05, ^*∗∗*^ *P* < 0.01.

There has been no previous report on the target and mechanism of regulation miRNA expression by QSYQ on ischemic CMECs angiogenesis. In this study, we used miRNA chip and real-time PCR techniques to analyze the optimum QSYQ dose for regulation of miRNA expression during ischemic CMEC angiogenesis. Compared with N group, there were 8 upregulated and 15 downregulated miRNAs in M group (Table 2) and 16 upregulated and 18 downregulated miRNAs in Q-L group (Table 2). Compared with M group, there were 26 upregulated miRNAs and 21 downregulated miRNAs in Q-L group (Table 2). Considering the link between differences in miRNA expression and angiogenesis, mir-223-3p was identified as the key miRNA during the proliferation period in the Q-L group. Compared with M group, the expression of mir-223-3p was downregulated 0.117 times in Q-L group, which showed a significant expression difference (*P* < 0.01). Thus, we concluded that mir-223-3p was the core miRNA for regulating ischemic CMECs angiogenesis by QSYQ. The miRNA expression heat map is presented in Figure 5(a).

Real-time PCR was used to verify the results from miRNA chip experiments on mir-223-3p expression and the results were found to be consistent. Compared with N group, there was significant upregulation of mir-223-3p expression in M group (*P* < 0.01) and downregulation in Q-L group (*P* < 0.05); compared with M group, there was significant downregulation of mir-223-3p in Q-L group (*P* < 0.01, Figure 5(b)).

### 3.5. The mRNA and Protein Expression of Target Gene* RPS6KB1*

**Figure 6:**
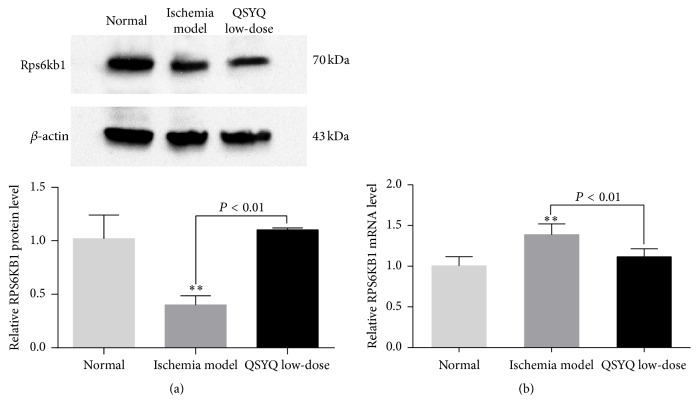
(a) Western-blot analyzed the protein level of RPS6KB1 in Q-L group and M group as compared to N group. It was showed that there was no significant difference in Q-L group and N group. *β*-actin was used as internal controls. ^*∗∗*^ *P* < 0.01. (b) Real-time PCR analyzed the mRNA level of RPS6KB1 in Q-L group and M group as compared to N group. It was showed that there was no significant difference in Q-L group and N group. ΔCt values were normalized to GAPDH levels. ^*∗∗*^ *P* < 0.01.

miRNAs play a role through regulating expression of target gene. It had been confirmed in previous study that* Rps6kb1* was the target gene of mir-223-3p in regulating angiogenesis of ischemic CMECs. The results of* Rps6kb1* in western-blot analysis demonstrated that, compared with N group, there was significant downregulation in M group (*P* = 0.001) and upregulation in Q-L group (*P* = 0.491); compared with M group, there was significant upregulation in Q-L group (*P* = 0.001, Figure 6(a)). The results of mRNA expression of* Rps6kb1* demonstrated that, compared with N group, there was significant upregulation in M group (*P* = 0.000) and upregulation in Q-L group (*P* = 0.051); compared with M group, there was significant downregulation in Q-L group (*P* = 0.000, Figure 6(b)). The segregation between mRNA and protein expression of target gene* Rps6kb1* was consistent with the mechanism of miRNA negative regulation, while there was a significant improvement after QSYQ intervention.

### 3.6. QSYQ Promote Ischemic CMECs Angiogenesis by Regulating mir-223-3p Expression, Possibly Related to Upregulation of RPS6KB1/HIF-1*α* Signaling Pathway

**Figure 7:**
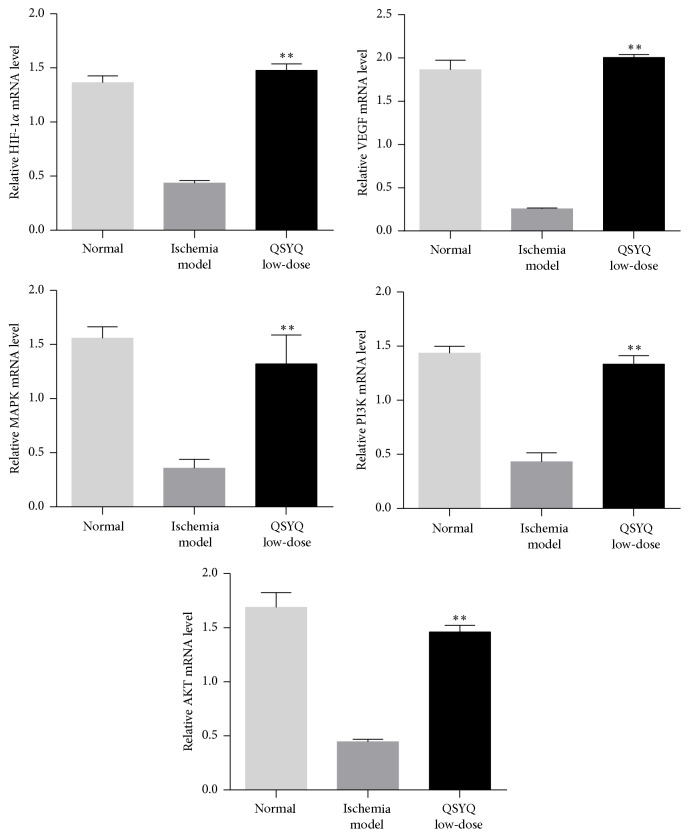
Real-time PCR analyzed the mRNA level of HIF-1*α*, VEGF, MAPK, PI3K, and AKT in Q-L group compared with M group. ^*∗∗*^ *P* < 0.01.

**Figure 8:**
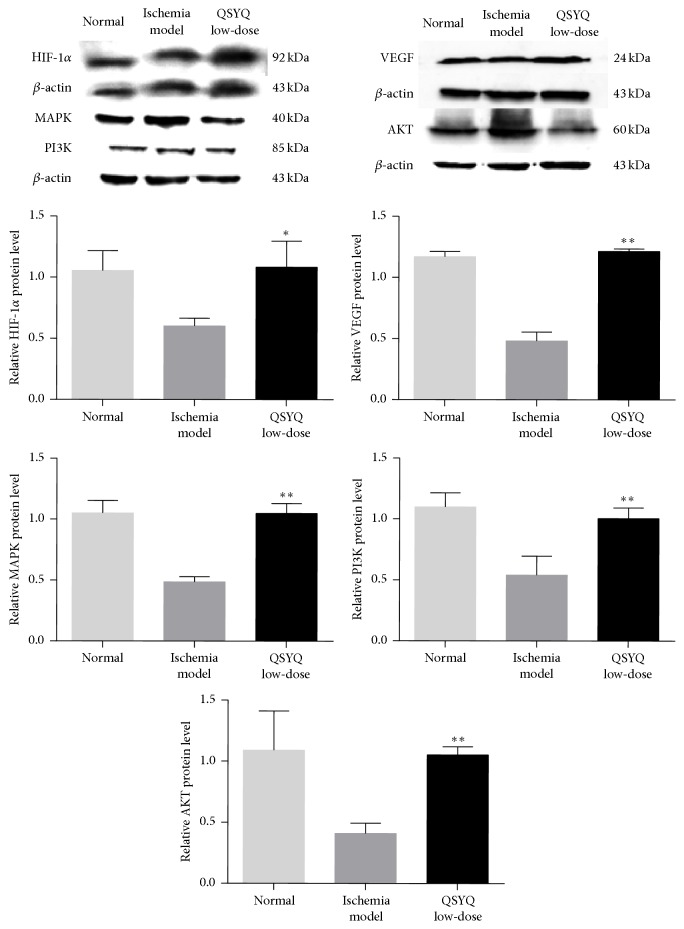
Western-blot analyzed the protein level of HIF-1*α*, VEGF, MAPK, PI3K, and AKT in Q-L group compared with M group. *β*-actin was used as internal controls. ^*∗*^ *P* < 0.05, ^*∗∗*^ *P* < 0.01.

Previous research showed that HIF-1*α* was an important transcription regulating factor for VEGF [15] and angiogenesis could be induced by upregulation of VEGF mRNA expression [16]. To further understand the mechanism of regulation of mir-223-3p expression by QSYQ in ischemic CMECs angiogenesis, we analyzed the signaling pathway of predicted RPS6KB1/HIF-1*α* and used real-time PCR and western-blot to test mRNA and protein expression, respectively, of related molecules in the signaling pathway: HIF-1*α*, VEGF, MAPK, PI3K, and AKT. Results showed that, compared with M group, mRNA and protein expression of the above-mentioned molecules in the Q-L group were significantly elevated (*P* < 0.01 or *P* < 0.05, Figures 7 and 8), suggesting that QSYQ induced expression of related molecules on the RPS6KB1/HIF-1*α* signaling pathway via downregulating mir-223-3p expression during CMECs angiogenesis and thereby promoted ischemic cardiac angiogenesis.

## 4. Discussions

Previous research showed that QSYQ can elevate mRNA and protein expression of VEGF and basic fibroblast growth factor, promote angiogenesis, increase blood supply in ischemic areas, reduce myocardial infarction area, and function to combat cardiac ischemia [17]. Previous studies also showed that miRNAs are closely associated with angiogenesis and they promote/inhibit angiogenesis by regulating target gene expression [18, 19]. However, there is no detailed report to date on the regulation of miRNAs by QSYQ.

The previous experiment results showed that mir-223-3p was a direct target of rats ischemic CMECs angiogenesis, decreased expression of VEGF, MAPK, PI3K, and so forth, inhibited proliferation and migration of ischemic CMECs, via affecting RPS6KB1/HIF-1*α* signal pathway, and thereby suppressed angiogenesis of ischemic myocardium. Based on previous study and the results, this study figured out the optimum dosage and window period for QSYQ for the regulation of ischemic CMECs angiogenesis. The present study confirmed that QSYQ could promote the angiogenesis of ischemic myocardium, though it did not demonstrate a dose-dependent style. The results indicated that the proliferation window period of ischemic CMECs in Q-L group was four days ahead of that of the M group and the migration and tube formation rate of Q-L group were significantly higher than other groups (*P* = 0.000), which suggested that Q-L group and proliferation period were the optimum dosage and window period for QSYQ intervening in ischemic CMECs angiogenesis. To further explore the function target and mechanisms of QSYQ in the regulation of ischemic CMECs angiogenesis based on miRNA, we used the optimum dosage and window period of QSYQ as the intervention group, used gene chip to analyze the miRNA active expression changes from intervention group, M group, and N group, and found that expression of mir-223-3p in intervention group was downregulated 0.117 times as much as M group, denoting a very significant difference. Real-time PCR analysis on mir-223-3p agreed with chip results. After comprehensive analysis, we confirmed mir-223-3p as the core miRNA from QSYQ in regulating ischemic CMECs angiogenesis. The results of mRNA and protein expression of* Rps6kb1* as the target of mir-223-3p demonstrated the segregation phenomenon between them, which was consistent with the mechanism of miRNA negative regulation. Following that, we found that there was an obvious improvement after QSYQ intervention.

The miRNAs function through regulating target gene expressions. To further discuss the mechanism of the regulation on mir-223-3p after the intervention from QSYQ, we tested related molecules HIF-1*α*, VEGF, MAPK, PI3K, and AKT with real-time PCR and western-blot. Results showed significant upregulation from the mRNA and protein expressions of the above-mentioned molecules. VEGF, an important regulator of angiogenesis, which is usually induced to upregulate by hypoxia and ischemia, promotes angiogenesis [20]. MAPK can activate a variety of transcription factors, promotes gene expression, and further promotes cell proliferation and differentiation. PI3K is a core signaling molecule of human life activities, and the mediated signal transduction pathway can regulate cell proliferation, differentiation, apoptosis, and other processes [21, 22]. AKT, a key factor for cell survival, plays an important role in antiapoptosis, cell survival, and proliferation. A number of studies indicate that many growth factors induced by hypoxia can increase the proliferation of vascular smooth muscle cells via PI3K/AKT signaling pathway, and MAPK, as a target molecule of PI3K downstream, can regulate cell proliferation by PI3K/MAPK signaling pathway. In conclusion, the study suggests that QSYQ can promote angiogenesis by downregulating mir-223-3p expressions and elevating expressions of factors on the RPS6KB1/HIF-1*α* signaling pathway, including VEGF, MAPK, and PI3K.

QSYQ are the main representative of Qi-replenishing and blood-activating TCM to treat ischemic heart disease, with several positive treatment features. Research shows that all components of QSYQ promote angiogenesis and improve cardiac ischemia [23–26]. Astragaloside IV, one of the main components from* Astragalus*, promotes angiogenesis primarily through activating PI3K/AKT signaling pathway and promoting HIF-1*α* protein synthesis, thereby elevating VEGF mRNA levels [23]; the main component from red sage, tanshinone IIA, can elevate expressions of VEGF through increasing HIF-1*α* mRNA expressions [24], and red sage plus its various extracts can all promote angiogenesis, reduce myocardial infarction area, and improve heart functions; the effective component from* Panax notoginseng*, notoginsenoside, can promote angiogenesis via VEGF-KDR/FLK-1 pathway and PI3K-AKT-eNOS signaling pathway [25]; and* Dalbergia odorifera* has anticardiac ischemia functions as well [26]. The multitarget and multipathway regulation characteristics of TCM components function well in various treatment modalities. Therefore, new ideas for TCM and miRNA research have been provided from discussion of the link between QSYQ and miRNAs.

Our study found via gene chip analysis that QSYQ can regulate multiple miRNA expression and intervention with ischemic CMECs angiogenesis. Among these, mir-223-3p showed the most significant expression changes, suggesting that QSYQ promote ischemic cardiac angiogenesis through regulating mir-223-3p expressions, which also involves upregulation of RPS6KB1/HIF-1*α* signaling pathway, and these have provided experimental evidence for research on miRNA array analysis of ischemic CMECs and drug intervention. Nonetheless, our current results are only limited to experimental research, more rigorous, and thorough clinical studies are needed in the future to provide more robust theoretical evidence for TCM in treating ischemic heart disease.

## Figures and Tables

**Table 1:** The window phases of proliferation, migration, and tube formation of the five groups.

Group	N	M	Q-H	Q-M	Q-L
Proliferation	The third day	The sixth day	The third day	The second day	The second day
Migration	Half of a day	Half of a day	Half of a day	Half of a day	Half of a day
Tube formation	The second day	The second day	The second day	The second day	The second day

**Table 2:** The differential expression of miRNAs in ischemia model group, QSYQ low-dose group, and normal group.

Gene	Fold change	*P* value	Gene	Fold change	*P* value
Upregulated miRNAs in ischemia model versus normal group					
rno-miR-346	96.774	0.014	rno-miR-511-3p	3.294	0.000
rno-miR-142-5p	4.741	0.000	rno-miR-146a-5p	2.395	0.000
rno-miR-142-3p	4.533	0.000	rno-miR-155-5p	2.171	0.000
rno-miR-223-3p	4.299	0.000	rno-miR-494-5p	2.056	0.002

Downregulated miRNAs in ischemia model versus normal group					
rno-miR-3596a	0.490	0.028	rno-miR-675-5p	0.415	0.042
rno-miR-802-5p	0.486	0.040	rno-miR-132-5p	0.392	0.000
rno-miR-140-5p	0.480	0.005	rno-miR-409b	0.383	0.008
rno-miR-132-3p	0.460	0.000	rno-miR-3596b	0.372	0.008
rno-miR-3560	0.447	0.004	rno-miR-300-3p	0.340	0.017
rno-miR-140-3p	0.440	0.001	rno-miR-10a-3p	0.337	0.035
rno-miR-330-3p	0.440	0.049	rno-miR-139-5p	0.312	0.003
rno-miR-212-3p	0.429	0.005			

Upregulated miRNAs in QSYQ low-dose versus normal group					
rno-miR-205	5.660	0.005	rno-miR-130b-5p	2.498	0.031
rno-miR-802-5p	5.562	0.043	rno-miR-551b-5p	2.279	0.048
rno-miR-539-3p	4.205	0.012	rno-miR-370-5p	2.198	0.006
rno-miR-3560	4.052	0.035	rno-miR-547-5p	2.156	0.013
rno-miR-93-3p	3.020	0.016	rno-miR-203a-3p	2.135	0.009
rno-miR-3549	2.656	0.025	rno-miR-291a-3p	2.039	0.030
rno-miR-222-3p	2.538	0.000	rno-let-7b-3p	2.034	0.024
rno-miR-760-5p	2.509	0.042	rno-miR-221-3p	2.008	0.000

Downregulated miRNAs in QSYQ low-dose versus normal group					
rno-miR-186-3p	0.490	0.016	rno-miR-668	0.434	0.002
rno-miR-195-5p	0.484	0.007	rno-miR-300-3p	0.404	0.024
rno-miR-412-3p	0.471	0.008	rno-miR-221-5p	0.388	0.021
rno-miR-665	0.463	0.002	rno-miR-342-5p	0.371	0.038
rno-miR-336-5p	0.460	0.020	rno-miR-224-5p	0.340	0.003
rno-miR-674-3p	0.458	0.015	rno-miR-1843-5p	0.310	0.004
rno-miR-15b-5p	0.457	0.006	rno-miR-154-3p	0.303	0.003
rno-miR-342-3p	0.441	0.006	rno-miR-511-3p	0.254	0.001
rno-miR-212-3p	0.440	0.011	rno-miR-363-5p	0.192	0.001

Upregulated miRNAs in QSYQ low-dose versus ischemia model group					
rno-miR-802-5p	11.437	0.031	rno-miR-547-5p	2.944	0.007
rno-miR-3560	9.074	0.020	rno-miR-192-5p	2.760	0.049
rno-miR-30c-1-3p	7.638	0.049	rno-miR-203a-3p	2.718	0.005
rno-miR-138-1-3p	6.987	0.036	rno-miR-200c-5p	2.622	0.022
rno-miR-205	6.898	0.004	rno-miR-25-5p	2.600	0.039
rno-miR-539-3p	5.222	0.012	rno-miR-505-5p	2.497	0.040
rno-miR-878	4.879	0.037	rno-miR-652-5p	2.487	0.035
rno-miR-93-3p	4.862	0.010	rno-miR-376b-3p	2.311	0.005
rno-miR-760-5p	4.789	0.018	rno-miR-410-5p	2.278	0.019
rno-miR-3549	4.685	0.009	rno-miR-23a-5p	2.209	0.019
rno-miR-743b-5p	4.681	0.034	rno-miR-134-3p	2.147	0.016
rno-miR-370-5p	3.662	0.003	rno-miR-299b-5p	2.134	0.038
rno-miR-132-5p	3.089	0.002	rno-miR-204-3p	2.125	0.019

Downregulated miRNAs in QSYQ low-dose versus ischemia model group					
rno-miR-350	0.495	0.003	rno-miR-142-3p	0.300	0.000
rno-miR-338-5p	0.490	0.002	rno-miR-221-5p	0.279	0.008
rno-miR-154-3p	0.453	0.003	rno-miR-1843-5p	0.274	0.006
rno-miR-532-5p	0.435	0.002	rno-miR-146a-5p	0.265	0.000
rno-miR-336-5p	0.425	0.005	rno-miR-142-5p	0.210	0.000
rno-miR-15b-5p	0.417	0.006	rno-miR-363-5p	0.184	0.033
rno-miR-668	0.412	0.021	rno-miR-1843-5p	0.151	0.021
rno-miR-339-5p	0.396	0.000	rno-miR-223-3p	0.117	0.000
rno-miR-380-5p	0.374	0.031	rno-miR-511-3p	0.077	0.000
rno-miR-342-5p	0.369	0.029	rno-miR-346	0.074	0.018
rno-miR-674-3p	0.313	0.001			
